# Proximity ligation assay to detect DUX4 protein in FSHD1 muscle: a pilot study

**DOI:** 10.1186/s13104-022-06054-8

**Published:** 2022-05-10

**Authors:** Mary Lou Beermann, Sachiko Homma, Jeffrey Boone Miller

**Affiliations:** Department of Neurology, Boston University School of Medicine, 700 Albany Street, Room 408K, Boston, MA 02118 USA

**Keywords:** DUX4, Facioscapulohumeral muscular dystrophy, FSHD, Skeletal muscle biopsy, Proximity ligation assay

## Abstract

**Objective:**

Aberrant expression in skeletal muscle of DUX4, a double homeobox transcription factor, underlies pathogenesis in facioscapulohumeral muscular dystrophy (FSHD). Although previous studies of FSHD muscle biopsies detected mRNAs encoding DUX4 and its target genes, no studies had reported detection of DUX4 protein. Our objective was to develop a proximity ligation assay (PLA) for DUX4 and to determine if this assay could detect DUX4 protein in FSHD muscle sections.

**Results:**

We developed a PLA protocol using two DUX4 antibodies previously reported by Stephen Tapscott’s group: P2G4, a mouse mAb specific for an epitope in the N-terminal region, and E5-5, a rabbit mAb specific for an epitope in the C-terminal region, in combination with commercial PLA secondary reagents. We validated the DUX4 PLA using cultured human myogenic cells in which DUX4 was ectopically expressed in a small fraction of nuclei. Using this two primary mAb PLA on an FSHD1 biceps biopsy, we observed nuclei with apparent DUX4 PLA signals associated with a small subset of myofibers (~ 0.05–0.1%). Though a limited pilot study, these results suggest that the two primary mAb PLA protocol could be useful for detecting DUX4 protein in FSHD muscle biopsies.

**Supplementary Information:**

The online version contains supplementary material available at 10.1186/s13104-022-06054-8.

## Introduction

Aberrant expression in skeletal muscle of DUX4, a double homeobox transcription factor, underlies pathogenesis in facioscapulohumeral muscular dystrophy (FSHD) [1–5]. In the two forms of the disease—FSHD1 (~ 95% of cases) and FSHD2 (~ 5% of cases)—DUX4 is expressed from an open reading frame in the most telomeric 3.3 kb D4Z4 repeat on chromosome 4q [6–8], with rare cases transcribed from chromosome 10q [9]. In differentiated cultures of myogenic cells obtained from FSHD1 patients, DUX4 protein can be detected by immunostaining in only a small fraction of myotube nuclei, typically 0.01–0.1% [7, 10–15]. In FSHD muscle biopsies, low levels of DUX4 mRNA, as well as mRNAs encoding DUX4 target genes, have been detected [16]. However, no previous studies had reported detection of DUX4 protein in FSHD muscle biopsies. The objectives of this pilot study, therefore, were to (i) develop and validate in cells a proximity ligation assay (PLA) [17–19] for DUX4 and (ii) determine if this assay could detect DUX4 protein in an FSHD1 muscle biopsy. We used PLA because of the high specificity and low background that can be achieved with this technique [17–19]. The results, though limited and requiring replication and expansion, suggest that PLA could be of interest as a useful technique to detect DUX4 protein in sections of biopsied FSHD muscle.

## Main text

### Methods and materials

#### Cells

Human primary myogenic cells (21Ubic) were from an unaffected donor, and previous publications have described institutional approvals, isolation, culture conditions, and authentication protocols for these cells [11–13, 15, 20]. Also see Declarations.

#### Biopsies

The FSHD1 biceps biopsy (#6524) was from a 17 year-old male donor with a very short 16 kb (~ 5 repeats), D4Z4 length. The unaffected biceps biopsy (#9557) was from a 33 year-old female donor. Biopsies were provided by Dr. Marina Mora and Dr. Maurizio Moggio (Fondazione IRCCS Ca' Granda Ospedale Maggiore Policlinico, Milan, Italy) through the Telethon Network of Genetic Biobanks and were maintained frozen at − 80 °C until sectioned. As noted further under Declarations, biopsies were collected under approved institutional protocols.

#### BacMam vectors

Two mammalian baculovirus (BacMam) vectors were used. To express full-length DUX4 tagged with a C-terminal V5 epitope in myoblasts, we used a modified mammalian baculovirus, termed BacMam-DUX4-V5, as described [13, 15, 21, 22]. As a negative control, we infected parallel cultures with a separate mammalian baculovirus, termed BacMam-GFP [13, 15], which leads to ectopic expressed of GFP. For both baculovirus vectors expression of the ectopic protein was under control of the human CMV-IE1 promoter. The amount of BacMam-DUX4-V5 or BacMam-GFP added to cultures was adjusted so that expression of DUX4 or GFP occurred in ~ 1–2% of the cells in cultures of human primary myoblasts.

#### Antibodies

Two DUX4 antibodies, both previously generated by Geng et al. [23], were used in this study: (i) P2G4 (a gift of Dr. Stephen Tapscott) which is a mouse mAb specific for an epitope in the N-terminal region; and (ii) E5-5 (cat. ab124699, Abcam, Boston MA), which is a rabbit mAb specific for an epitope in the C-terminal region. Immunostaining for the V5 epitope or GFP were as described [13, 15, 22, 24].

#### Preparation of cells for proximity ligation assay

Undifferentiated 21Ubic myoblasts were grown on porcine gelatin-coated, 4-well permanox chamber slides (cat.177437, Thermo Fisher, Waltham MA) and incubated for 48 h with BacMam-DUX4-V5 or BacMam-GFP, after which the cultures were fixed in 2% paraformaldehyde in PBS for 10 min at room temperature [13, 15]. After three 5 min washes with PBS, PBS plus 10 mM glycine, and PBS, the cultures were quickly rinsed with deionized water. The fixed cultures were permeabilized in 0.5% Triton X-100 in PBS and then incubated for 30 min at 37 °C in blocking solution, which consisted of 4% normal goat serum (Vector Labs, Burlingame CA), 4% normal horse serum (Vector Labs), 4% bovine serum albumin (Sigma-Aldrich, St. Louis MO), and 0.1% Triton X-100 (Sigma-Aldrich) in PBS. The fixed and blocked cells were incubated overnight at 4 °C with both the N-terminal P2G4 mouse mAb and the C-terminal E5-5 rabbit mAb, each diluted 1:250 and mixed together in blocking solution.

#### Preparation of muscle sections for proximity ligation assay

Frozen biopsies were embedded in Sakura Tissue-Tek O.C.T. (cat. 27050, Ted Pella Inc. Redding CA), and, using a cryostat, 10 µm serial sections were made from each biopsy and collected on ten glass slides at three sections per slide. Slides were air-dried for 30 min and each section was encircled with a Pap Pen (cat. H4000, Vector Labs) to make a well for reagents. Encircled sections were rehydrated in PBS for 10 min, fixed in ice-cold 100% methanol for 10 min, washed for three times at 5 min each with PBS, washed once quickly with deionized water, and then incubated in blocking solution for 30 min at 37 °C. The fixed and blocked muscle sections were incubated overnight at 4 °C with both the N-terminal P2G4 mouse mAb and the C-terminal E5-5 rabbit mAb, each diluted 1:250 and mixed together in blocking solution.

#### Proximity ligation assay

After overnight incubation at 4 °C with the mixture of mouse P2G4 and rabbit E5-5 mAbs, cells or sections were washed and processed for PLA exactly according to the manufacturer’s published protocols [25, 26]. PLA reagents, all from Sigma-Aldrich (St. Louis MO), included the following:

1. DUO92004. Duolink In Situ PLA Probe Anti-Mouse MINUS, Affinity purified Donkey anti-Mouse IgG (H + L) (lot A40108).
2. DUO92002. Duolink In Situ PLA Probe Anti-Rabbit PLUS, Affinity purified Donkey anti-Rabbit IgG (H + L) (lot A40406).
3. DUO92008. Duolink In Situ Detection Reagents Red. This reagent kit included (i) 5× Ligation, which contained oligonucleotides that hybridized to the PLA probes and components needed for ligation; (ii) 1× Ligase (1 unit/μL); (iii) 1× Polymerase (10 units/μL); (iv) 5× Amplification Red, which contained components needed for rolling circle amplification and oligonucleotide probes labeled with red fluorophore that hybridized to the amplification product.
4. DUO82049. Duolink in situ wash buffers A and B.
5. DUO82040. Duolink in situ mounting medium with DAPI.

Images of cells and sections were acquired using a 20× or 40× objective on a Nikon E800 microscope equipped with a Spot camera and software version 5.1 (Diagnostic Instruments Inc., Sterling Heights, Michigan).

### Results and discussion

**Fig. 1:**
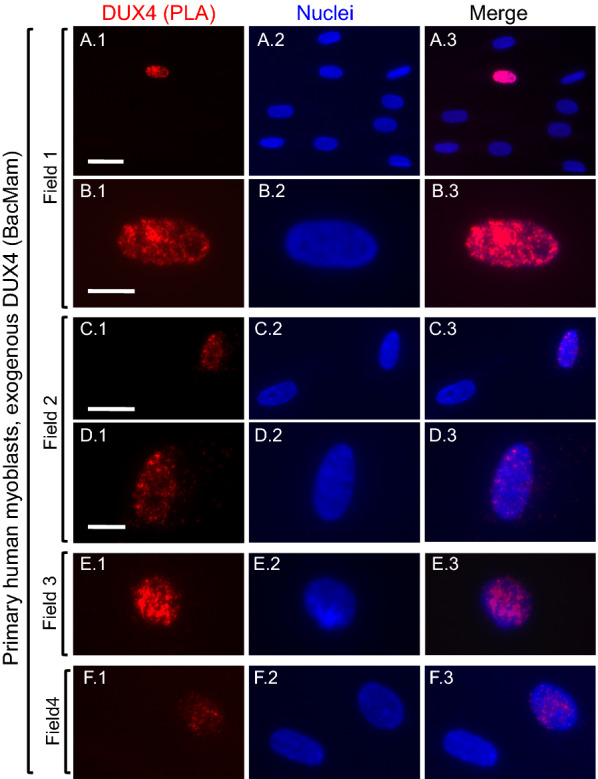
Validation of proximity ligation assay (PLA) for DUX4. Cultures of unaffected, primary human myoblasts were incubated with BacMam-DUX4 at a multiplicity of infection that generated DUX4 expression in a small proportion of the myoblasts. At 48 h after BacMam addition, cultures were processed for two primary antibody PLA (red signal) as described in Methods. Row **A** shows a single nucleus with a positive PLA signal (red) amidst eight nuclei that were unstained; and Row **B** shows the same nucleus at higher magnification to emphasize the characteristic punctate staining pattern expected for a PLA signal. Nuclei were stained with bisbenzimide (blue). Row **C** shows one nucleus with a positive PLA signal and a nearby unstained nucleus; and row **D** shows the positive nucleus at higher magnification. Rows **E** and **F** show additional examples of nuclei with positive PLA signals. Bar in **A**1 = 30 µm for row A. Bar in **B **1 = 10 µm for row **B**. Bar in **C**1 = 25 µm for row **C**. Bar in **D** 1 = 10 µm for rows **D**, **E**, and **F**

We first used the two mAb PLA protocol (as described in Methods) on primary human myoblasts that had been incubated for 48 h with an amount of BacMam-DUX4-FL-V5 that, based on V5 immunocytochemistry, generated DUX4 expression in a small percentage (~ 1–2%) of the host cells. In such cultures, we found a similarly small percentage of myoblast nuclei that showed a positive PLA signal for DUX4 (Fig. 1 and Additional file 1: Fig. S1). These nuclear signals had the characteristic pattern of small fluorescent puncta expected for PLA [17, 18]. Background staining (i.e., non-nuclear) was almost absent (Fig. 1 and Additional file 1: Fig. S1). The PLA signal was inhomogeneously distributed within most nuclei, with different regions showing lower or higher densities of PLA puncta. The degree of intra-nuclear signal inhomogeneity varied among nuclei (e.g. compare Fig. 1B–F). Standard immunofluorescence assays with mAb E5-5 also show inhomogeneity in DUX4 distribution within many individual nuclei [13, 15, 24]. As controls, we noted that nuclear PLA signals were not found when (i) mouse mAb P2G4 was replaced with an anti-GFP antibody or (ii) the myoblasts were incubated with BacMam-GFP instead of BacMam-DUX4-FL-V5. Thus, the two primary mAb PLA protocol showed low background and high specificity for BacMam-mediated DUX4 protein expression in myoblasts.

Encouraged by these PLA results with myoblasts, we next used the two primary mAb PLA protocol on sections of FSHD1 and unaffected muscle biopsies (see Methods). Histological analyses of sections from the FSHD1 biopsy (Additional file 1: Fig. S2) showed minimal myopathic changes [27], including a small percentage of angular (possibly atrophic) myofibers, as well as occasional central nucleate myofibers. There was little or no evidence of extensive fiber rounding, perivascular inflammation, or fatty replacement.

**Fig. 2:**
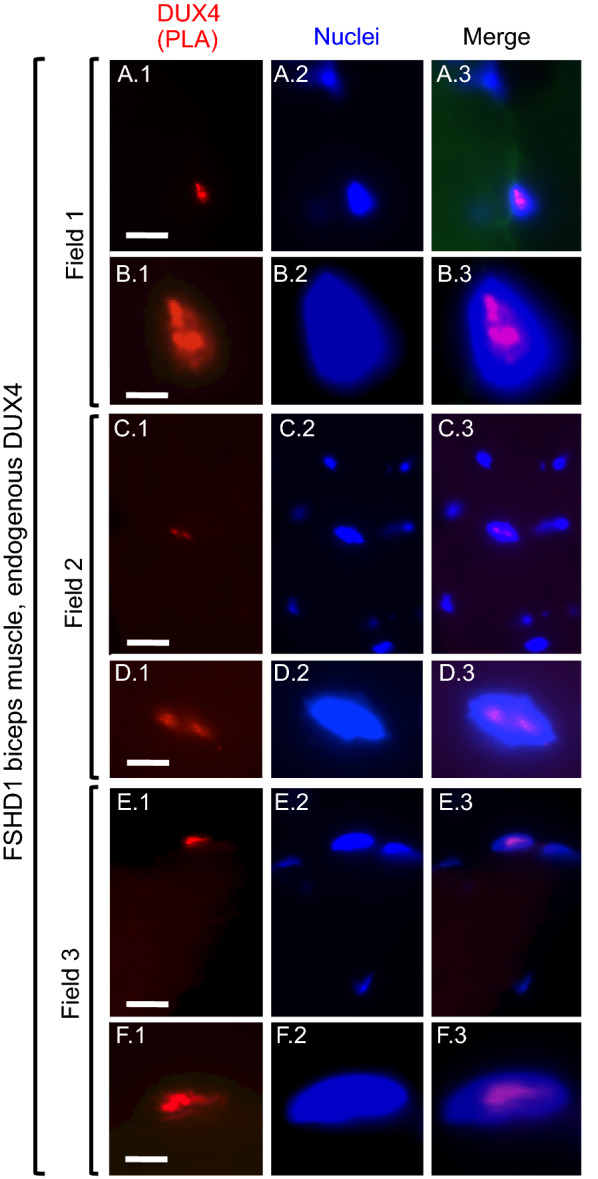
Nuclear DUX4 PLA signals in an FSHD1 biopsy (microscope fields 1–3). Sections of biceps muscle obtained from a FSHD1 donor were processed for two primary antibody PLA and nuclei were stained with bisbenzimide. As indicated, each row includes images of a single field showing the DUX4 PLA signal (red), nuclei (blue), and merged PLA and nuclei signals. For field 1, row **A** shows a single nucleus with a positive PLA signal (red), as well as one nearby nucleus that was unstained; and Row **B** shows the positive nucleus at higher magnification. For field 2, row **C** shows one nucleus with a positive PLA signal amid several nearby unstained nuclei; and row **D** shows the positive nucleus at higher magnification. For field 3, row **E** shows an additional example of a nucleus with a positive PLA signal with three nearby unstained nuclei; and row **F** shows the positive signal at higher magnification. Bar in **A** 1 = 10 µm for row A. Bar in **B**1 = 4 µm for row **B**. Bar in **C**1 = 10 µm for row C. Bar in **D** 1 = 4 µm for row D. Bar in **E** 1 = 10 µm for row E. Bar in F.1 = 3.3 µm for row F

**Fig. 3:**
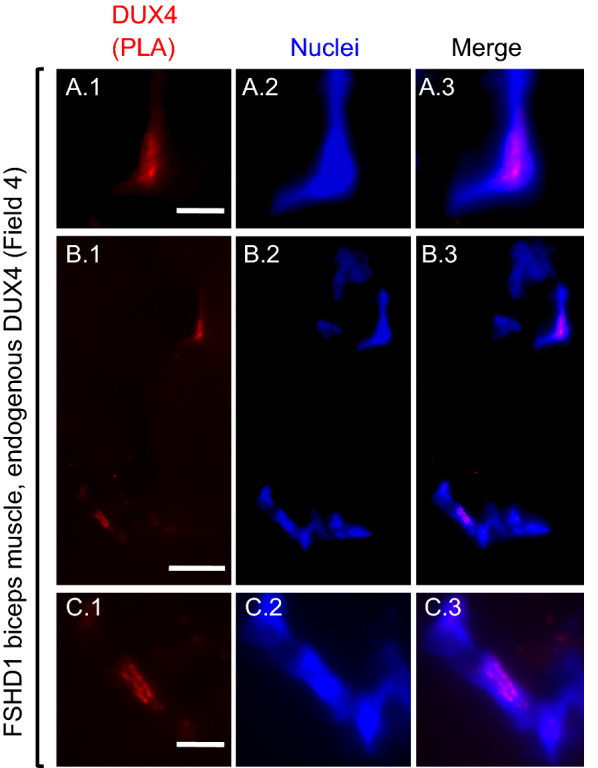
Nuclear DUX4 PLA signals in an FSHD1 biopsy (microscope field 4). As in Fig. 2, each row includes images from a single microscope field showing the DUX4 PLA signal (red), nuclei (blue), and merged PLA and nuclei signals. Images in row **A** include a nucleus-associated PLA signal (red); and this same region is also seen at lower magnification in the upper right corner of the images in row **B**. Images in row **B** show a lower magnification view of a field with two regions containing nucleus-associated PLA signals. Images in row **C** show at higher magnification the nucleus-associated PLA signal seen in the lower left corner of the row **B** images. Bar in **A** 1 = 5 µm for row **A**. Bar in **B** 1 = 15 µm for row **B**. Bar in **C** 1 = 5 µm for row **C**

When examining PLA signals on sections of this FSHD1 biceps muscle, we observed a small number of nuclei with PLA signals that were well above background and entirely within the nuclear boundary (Figs. 2, 3). In some nuclei, the PLA signal was evidently composed of multiple puncta (e.g. Figs. 2D, 3C), though, in others, puncta in the PLA signal were not well-resolved. When compared to PLA of myoblasts (Fig. 1), the individual PLA puncta in sections were generally less-well resolved, perhaps due to the varied orientations and greater thickness of muscle nuclei vs. the nuclei in cultured myoblasts. Also, nuclei with positive PLA signals were located at different depths in the sections, so different image planes were needed to visualize puncta when more than one PLA-positive nucleus was present within a single 40X microscope field (Additional file 1: Fig. S3). The methanol fixation used for the muscle sections also may have limited resolution vs. the paraformaldehyde fixation used for myoblasts.

On sections analyzed by PLA, the non-nuclear signals were generally low, consisting of scattered individual puncta, diffuse fluorescence in myofibers damaged during sectioning, and, on a minority of myofibers, regions of the cell border (Additional file 1: Fig. S4). When we analyzed sections of the unaffected muscle biopsy by PLA, we observed similar non-nuclear background, but we did not observe any nuclei with PLA signals.

We enumerated myofibers and DUX4-positive nuclei on four sections and found that ~ 0.05–0.1% of the FSHD1 myofibers were associated with DUX4-positive nuclei (~ 8000 myofibers had 6 DUX4-positive nuclei).

The two primary mAb protocol we developed appears to have good specificity for DUX4 protein in nuclei coupled with a low background on both cultured myoblasts and muscle sections. In earlier preliminary work, we also attempted to analyze muscle sections with standard single (mAb E5-5) and double (mAbs E5-5 and P2G4) immunofluorescence, as well as with a variation of the PLA protocol that used only single primary mAb (E5-5) with two secondary mAbs. Despite rare detection of DUX4-positive nuclei, each of these alternatives was limited by much higher background, particularly at myofiber surfaces and along connective tissue, than was seen with the two primary mAb PLA protocol reported here. As described in the Limitations section, we recognize that this study needs to be extended and replicated. Although we are not able to continue these studies (due to P.I. retirement), we have presented our initial work both because detection of DUX4 protein in FSHD muscle samples has not previously been reported and because our PLA protocol could prove useful to other investigators in further studies of FSHD pathogenesis.

## Limitations

Additional experiments are needed to overcome the limitations of this n = 1 pilot study. In particular, to replicate and extend confidence in these initial results, the two primary mAb PLA should be carried out on biopsies from many additional FSHD and unaffected donors. Coupling DUX4 PLA and mRNA analyses of each biopsy could determine how closely correlated the number of DUX4-positive nuclei is to the level of DUX4 mRNA. In addition, positive results from PLA analyses of one or more of the mouse models in which human DUX4 is expressed [28–32] could increase confidence in specificity of the protocol. Co-staining the PLA sections for dystrophin by immunofluorescence could be used to determine if the DUX4-positive nuclei are located within myofibers. Future studies would also be strengthened by using confocal microscopy to increase resolution and to allow three-dimensional reconstruction of PLA signals within myonuclei.

## Supplementary Information

**Additional file 1.** Additional figures.

## Data Availability

All data generated or analyzed during this study are included in this published article and its Additional file 1.

## Abbreviations

PLA: Proximity ligation assay
FSHD: Facioscapulohumeral muscular dystrophy

## References

[1] Campbell AE, Belleville AE, Resnick R, Shadle SC, Tapscott SJ (2018). Facioscapulohumeral dystrophy: activating an early embryonic transcriptional program in human skeletal muscle. Hum Mol Genet.

[2] Himeda CL, Jones PL (2019). The genetics and epigenetics of facioscapulohumeral muscular dystrophy. Annu Rev Genomics Hum Genet.

[3] Greco A, Goossens R, van Engelen B, van der Maarel SM (2020). Consequences of epigenetic derepression in facioscapulohumeral muscular dystrophy. Clin Genet.

[4] Wang LH, Tawil R (2021). Current therapeutic approaches in FSHD. J Neuromuscul Dis.

[5] Banerji CRS, Zammit PS (2021). Pathomechanisms and biomarkers in facioscapulohumeral muscular dystrophy: roles of DUX4 and PAX7. EMBO Mol Med.

[6] Lemmers RJ, van der Vliet PJ, Klooster R, Sacconi S, Camaño P, Dauwerse JG, Snider L, Straasheijm KR, van Ommen GJ, Padberg GW, Miller DG, Tapscott SJ, Tawil R, Frants RR, van der Maarel SM (2010). A unifying genetic model for facioscapulohumeral muscular dystrophy. Science.

[7] Snider L, Geng LN, Lemmers RJ, Kyba M, Ware CB, Nelson AM, Tawil R, Filippova GN, van der Maarel SM, Tapscott SJ, Miller DG (2010). Facioscapulohumeral dystrophy: incomplete suppression of a retrotransposed gene. PLoS Genet.

[8] Lemmers RJ, Tawil R, Petek LM, Balog J, Block GJ, Santen GW, Amell AM, van der Vliet PJ, Almomani R, Straasheijm KR, Krom YD, Klooster R, Sun Y, den Dunnen JT, Helmer Q, Donlin-Smith CM, Padberg GW, van Engelen BG, de Greef JC, Aartsma-Rus AM, Frants RR, de Visser M, Desnuelle C, Sacconi S, Filippova GN, Bakker B, Bamshad MJ, Tapscott SJ, Miller DG, van der Maarel SM (2012). Digenic inheritance of an SMCHD1 mutation and an FSHD-permissive D4Z4 allele causes facioscapulohumeral muscular dystrophy type 2. Nat Genet.

[9] Lemmers RJLF, van der Vliet PJ, Blatnik A, Balog J, Zidar J, Henderson D, Goselink R, Tapscott SJ, Voermans NC, Tawil R, Padberg GWAM, van Engelen BG, van der Maarel SM (2021). Chromosome 10q-linked FSHD identifies *DUX4* as principal disease gene. J Med Genet.

[10] Tassin A, Laoudj-Chenivesse D, Vanderplanck C, Barro M, Charron S, Ansseau E, Chen YW, Mercier J, Coppée F, Belayew A (2012). DUX4 expression in FSHD muscle cells: how could such a rare protein cause a myopathy?. J Cell Mol Med.

[11] Jones TI, Chen JC, Rahimov F, Homma S, Arashiro P, Beermann ML, King OD, Miller JB, Kunkel LM, Emerson CP, Wagner KR, Jones PL (2012). Facioscapulohumeral muscular dystrophy family studies of DUX4 expression: evidence for disease modifiers and a quantitative model of pathogenesis. Hum Mol Genet.

[12] Himeda CL, Debarnot C, Homma S, Beermann ML, Miller JB, Jones PL, Jones TI (2014). Myogenic enhancers regulate expression of the facioscapulohumeral muscular dystrophy-associated DUX4 gene. Mol Cell Biol.

[13] Homma S, Beermann ML, Boyce FM, Miller JB (2015). Expression of FSHD-related DUX4-FL alters proteostasis and induced TDP-43 aggregation. Ann Clin Transl Neurol.

[14] Rickard AM, Petek LM, Miller DG (2015). Endogenous DUX4 expression in FSHD myotubes is sufficient to cause cell death and disrupts RNA splicing and cell migration pathways. Hum Mol Genet.

[15] Homma S, Beermann ML, Yu B, Boyce FM, Miller JB (2016). Nuclear bodies reorganize during myogenesis in vitro and are differentially disrupted by expression of FSHD-associated DUX4. Skelet Muscle.

[16] Wong CJ, Wang LH, Friedman SD, Shaw D, Campbell AE, Budech CB, Lewis LM, Lemmers RJFL, Statland JM, van der Maarel SM, Tawil RN, Tapscott SJ (2020). Longitudinal measures of RNA expression and disease activity in FSHD muscle biopsies. Hum Mol Genet.

[17] Söderberg O, Leuchowius KJ, Gullberg M, Jarvius M, Weibrecht I, Larsson LG, Landegren U (2008). Characterizing proteins and their interactions in cells and tissues using the. Methods.

[18] Greenwood C, Ruff D, Kirvell S, Johnson G, Dhillon HS, Bustin SA (2015). Proximity assays for sensitive quantification of proteins. Biomol Detect Quantif..

[19] Raykova D, Koos B, Asplund A, Gelléri M, Ivarsson Y, Danielson UH, Söderberg O (2016). Let there be light!. Proteomes..

[20] Homma S, Chen JC, Rahimov F, Beermann ML, Hanger K, Bibat GM, Wagner KR, Kunkel LM, Emerson CP, Miller JB (2012). A unique library of myogenic cells from facioscapulohumeral muscular dystrophy subjects and unaffected relatives: family, disease and cell function. Eur J Hum Genet.

[21] Mitsuhashi H, Ishimaru S, Homma S, Yu B, Honma Y, Beermann ML, Miller JB (2018). Functional domains of the FSHD-associated DUX4 protein. Biol Open..

[22] Mitsuhashi H, Homma S, Beermann ML, Ishimaru S, Takeda H, Yu BK, Liu K, Duraiswamy S, Boyce FM, Miller JB (2019). Efficient system for upstream mRNA trans-splicing to generate covalent, head-to-tail, protein multimers. Sci Rep.

[23] Geng LN, Tyler AE, Tapscott SJ (2011). Immunodetection of human double homeobox 4. Hybridoma.

[24] Masteika IF, Sathya A, Homma S, Miller BM, Boyce FM, Miller JB (2022). Downstream events initiated by expression of FSHD-associated DUX4: studies of nucleocytoplasmic transport, γH2AX accumulation, and Bax/Bak-dependence. Biol Open.

[25] Sigma-Aldrich Duolink user manual. https://www.sigmaaldrich.com/US/en/technical-documents/protocol/protein-biology/protein-and-nucleic-acid-interactions/duolink-fluorescence-user-manual. Accessed 7 Feb 2022.

[26] Sigma-Aldrich Duolink short protocol. https://www.sigmaaldrich.com/US/en/technical-documents/protocol/protein-biology/protein-expression/duolink-short-protocol. Accessed 7 Feb 2022.

[27] Wang LH, Tawil R (2016). Facioscapulohumeral dystrophy. Curr Neurol Neurosci Rep.

[28] Nunes AM, Ramirez M, Jones TI, Jones PL (2021). Identification of candidate miRNA biomarkers for facioscapulohumeral muscular dystrophy using DUX4-based mouse models. Dise Models Mech..

[29] Flores P, Schreier S, Ramirez M, Wuebbles RD, Burkin DJ, Bradley RK, Jones PL (2020). Transgenic mice expressing tunable levels of DUX4 develop characteristic facioscapulohumeral muscular dystrophy-like pathophysiology ranging in severity. Skelet Muscle.

[30] Bosnakovski D, Shams AS, Yuan C, da Silva MT, Ener ET, Baumann CW, Lindsay AJ, Verma M, Asakura A, Lowe DA, Kyba M (2020). Transcriptional and cytopathological hallmarks of FSHD in chronic DUX4-expressing mice. J Clin Invest.

[31] Mueller AL, O’Neill A, Jones TI, Llach A, Rojas LA, Sakellariou P, Stadler G, Wright WE, Eyerman D, Jones PL, Bloch RJ (2019). Muscle xenografts reproduce key molecular features of facioscapulohumeral muscular dystrophy. Exp Neurol.

[32] Krom YD, Thijssen PE, Young JM, den Hamer B, Balog J, Yao Z, Maves L, Snider L, Knopp P, Zammit PS, Rijkers T, van Engelen BG, Padberg GW, Frants RR, Tawil R, Tapscott SJ, van der Maarel SM (2013). Intrinsic epigenetic regulation of the D4Z4 macrosatellite repeat in a transgenic mouse model for FSHD. PLoS Genet.

