# Supplementary material for: Proximity ligation assay to detect DUX4 protein in FSHD1 muscle: a pilot study
Source: BMC Res Notes. 2022 May 10;15:163. doi: 10.1186/s13104-022-06054-8 (PMC9092897; doi:10.1186/s13104-022-06054-8)
Supplement: Supplementary file 1 — Additional file 1. Additional figures. [file 13104_2022_6054_MOESM1_ESM.pdf]

Supplemental data file for:

Proximity ligation assay to detect DUX4 protein in sectioned FSHD1 muscle: A pilot study

Mary Lou Beerman, Sachiko Homma, and Jeffrey Boone Miller*

Department of Neurology
Boston University School of Medicine
Boston, Massachusetts USA 02118

Contents:

Page 2. Supplemental Fig. S1

Page 3. Legend for Fig. S1

Page 4. Supplemental Fig. S2

Page 5. Legend for Fig. S2

Page 6. Supplemental Fig. S3 and legend

Page 7. Supplemental Fig. S4

Page 8. Legend for Fig. S4

Fig. S1. Beermann et al.

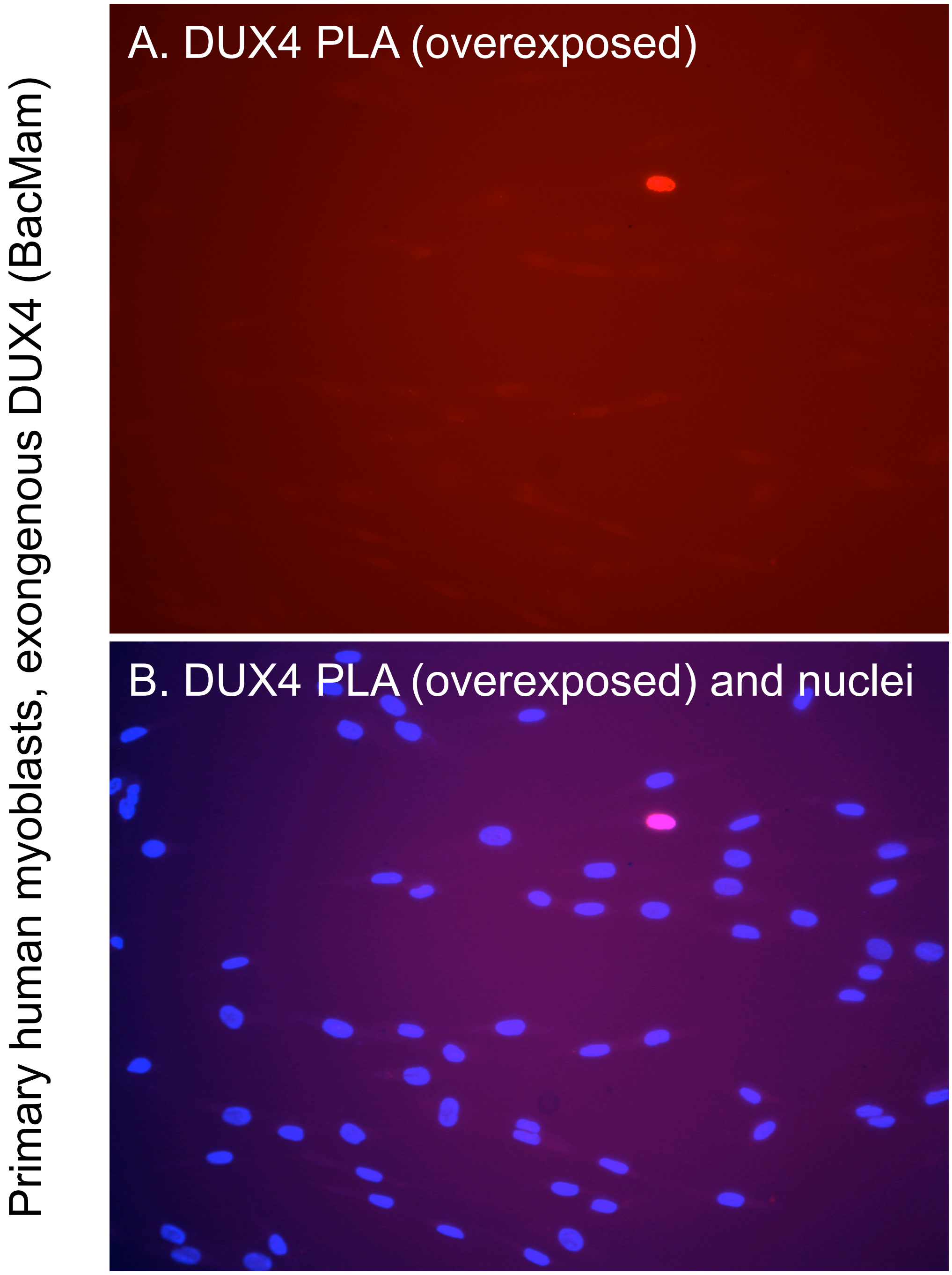

Fig. S1. **Low background with two mAb DUX4 PLA protocol used on human myoblasts**. As in Fig. 1, cultures of unaffected, primary human myoblasts were incubated with BacMam-DUX4-V5 at a multiplicity of infection that generated DUX4 expression in a small proportion (1–2%) of the myoblasts. At 48h after BacMam addition, cultures were processed for two antibody PLA (red signal) as described in Methods. **A.** Overexposure of 20X image shows a single nucleus with a positive DUX4 PLA signal (red) and very low background staining outside of that nucleus. Images of this single positive nucleus are shown at proper exposure and higher magnification in Figs. 1A and 1B. **B.** The image from panel A was merged with an image of the nuclei in the same field, showing that the DUX4 PLA protocol generated a signal in the expected small percentage of nuclei. Bar = 100 μm.

Fig. S2. Beermann et al.

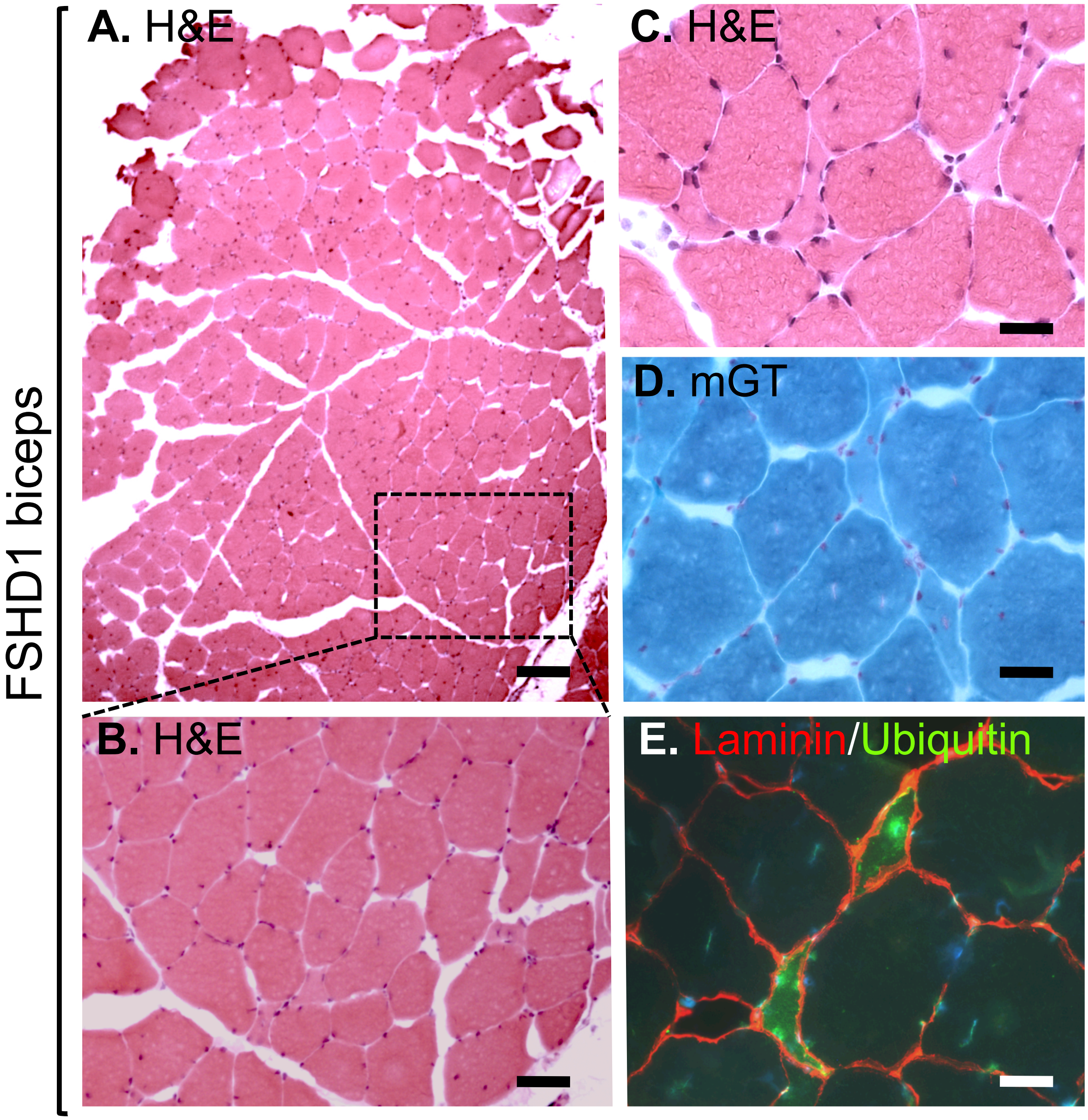

Fig. S2. **Histological analyses of FSHD1 biceps sections showed mild myopathic changes**. **A.** Hematoxylin and eosin (H&E) staining of a section of the FSHD1 biceps biopsy described in Methods. Adjacent sections were used for PLA. Bar = 250 µm. **B.** View of the boxed area from panel A. Bar = 90 µm. **C.** Higher magnification view of a different region of the FSHD1 biceps shows several smaller, angular (possibly atrophic) myofibers and some myofibers with centrally located nuclei. Bar = 50 µm. **D.** Modified Gomori trichrome (mGT) stain of an FSHD1 biceps section showing myopathic changes similar to those in panel C. Bar = 50 µm. **E.** Double immunohistofluorescence analysis of an FSHD1 biceps section. Laminin isoforms (red signal) were detected with a rabbit polyclonal antibody that reacts with multiple laminin subunits including laminins alpha-1, beta-1 and gamma-1 used at 1:50 dilution (Pan-laminin; L-9393; Sigma-Aldrich) coupled with goat anti-rabbit Alexa 594 (A11012; Invitrogen). Ubiquitinated proteins (green signal) were detected with mouse mAb FK2 (cat. D058-3, MBL International, Woburn, USA) used at 1:1000 coupled with goat anti-mouse Alexa 488 (A11001; Invitrogen); FK2 reacts with K29, K48, and K63 mono- and poly-ubiquitinated proteins, but not with free ubiquitin. Bar = 50 µm. We observed minimal myopathic changes in this FSHD1 biceps biopsy which included a small percentage of angular (possibly atrophic) myofibers, as well as occasional central nucleate myofibers. There was little or no evidence of extensive fiber rounding, perivascular inflammation, or fatty replacement.

Fig. S3. Beermann et al.

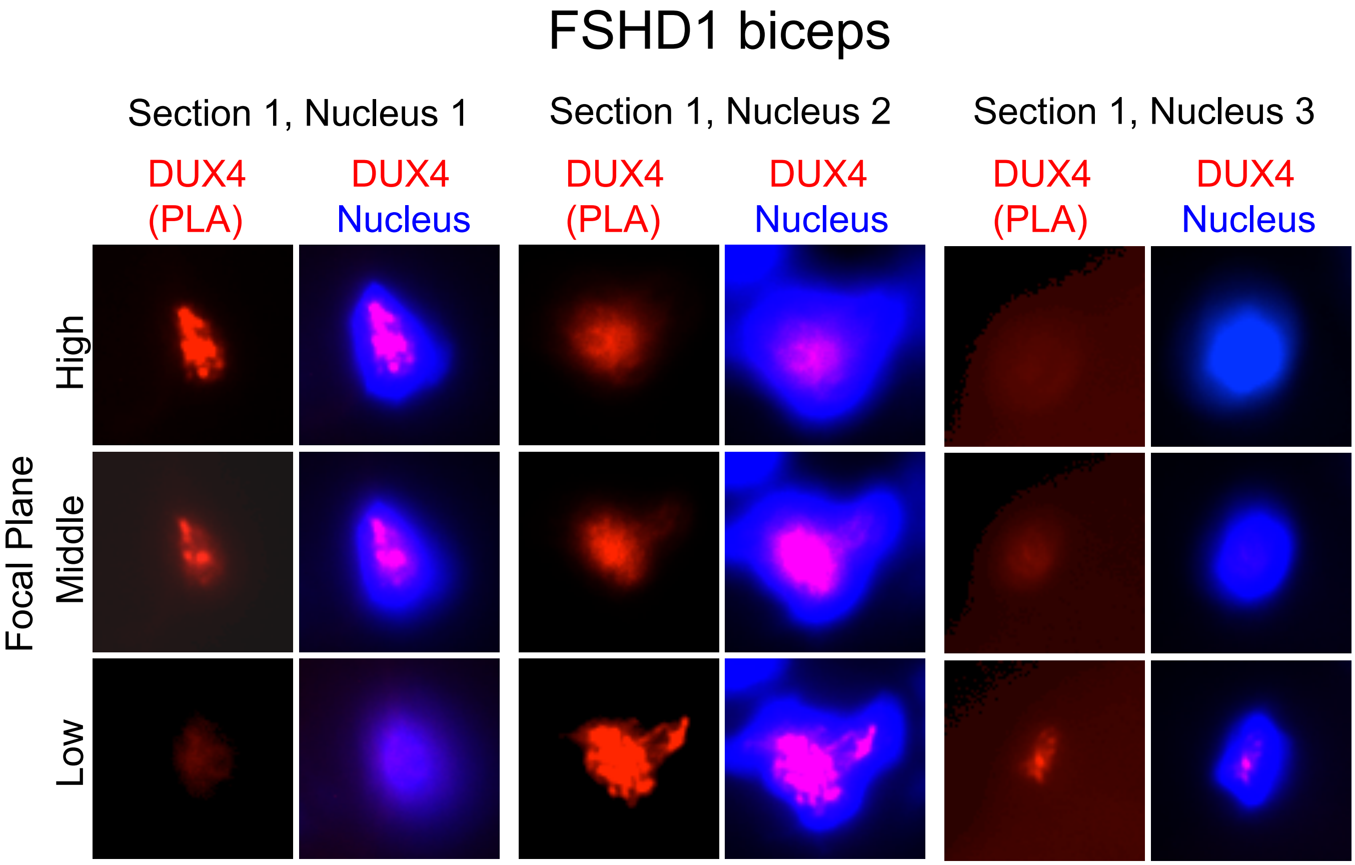

Fig. S3. Imaging at different focal planes of DUX4 PLA signals. DUX4 PLA signals associated with three different nuclei — all three of which were located within a single 40X field on a 10 µm thick section of FSHD1 biceps — were imaged at three different focal planes (low, medium, high) as indicated. In each nucleus, the PLA signal was most sharply resolved at the same depth as the nuclear borders were also sharpest. However, the PLA signals resolved at different focal planes for different nuclei, indicating that nuclei with positive PLA signals were located at different depths (i.e., along the z-axis) within a single 40X field (which measured 220 µm x 295 µm). Nucleus #1 is also shown in Fig. 1B. Bar = 5 µm.

Fig. S4. Beermann et al.

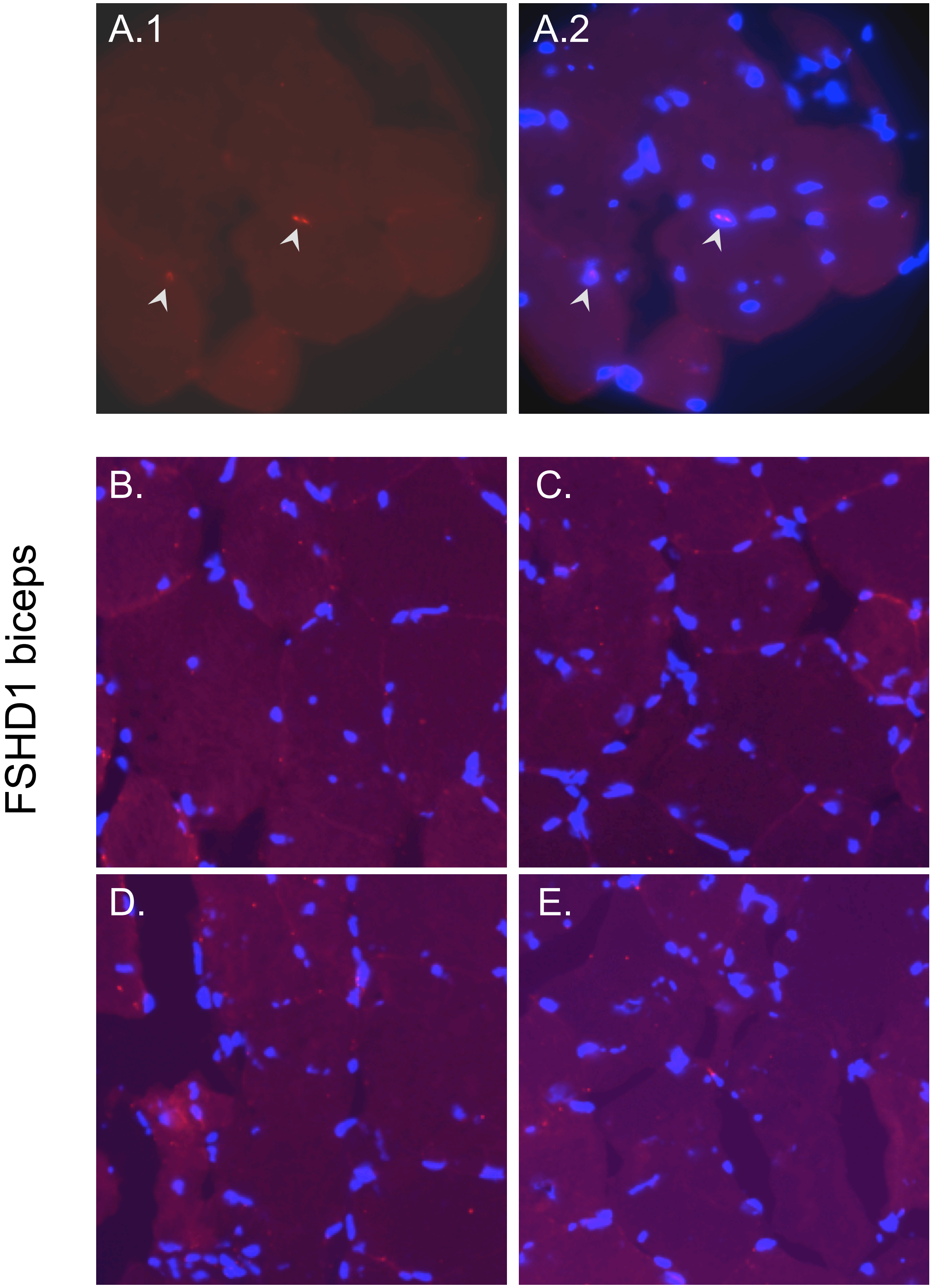

Fig. S4. **Examples of nuclear and non-nuclear red fluoresence on FSHD1 biceps sections analyzed by two mAb DUX4 PLA**. **A.** Region of an FSHD1 section prepared as described in Methods showing **A.1** DUX4 PLA signal (red) and **A.2.** DUX4 PLA plus nuclei (blue). The two arrows indicate nuclei containing PLA signals which appeared to be composed of multiple puncta contained entirely within the nuclear boundaries. Note that non-nuclear red fluorescence was much less intense and more scattered than the two signals associated with nuclei. The more centrally located of the two arrows points to a region that is shown at higher magnification in Figs. 2C and 2D. **B – E.** Four areas of an FSHD1 biceps muscle section showing the DUX4 PLA signal (red) and nuclei (blue). In these fields, none of the nuclei showed obvious PLA signals. Non-nuclear red fluorescence intensity was generally low, even on these overexposed images, though scattered individual puncta were seen and diffuse fluorescence was found at myofibers damaged during sectioning (e.g., as in panel **D**). In addition, a minority of myofibers showed regions of non-nuclear red fluorescence that appeared to be associated with the cell surface (e.g. as in panels **B**, **C**). Bar = 30μm.
